# Treatment of *β*-Thalassemia/Hemoglobin E with Antioxidant Cocktails Results in Decreased Oxidative Stress, Increased Hemoglobin Concentration, and Improvement of the Hypercoagulable State

**DOI:** 10.1155/2015/537954

**Published:** 2015-05-19

**Authors:** Orn-uma Yanpanitch, Suneerat Hatairaktham, Ratiya Charoensakdi, Narumol Panichkul, Suthat Fucharoen, Somdet Srichairatanakool, Noppadol Siritanaratkul, Ruchaneekorn W. Kalpravidh

**Affiliations:** ^1^Department of Biochemistry, Faculty of Medicine Siriraj Hospital, Mahidol University, Bangkok 10700, Thailand; ^2^Thalassemia Research Center, Institute of Molecular Biosciences, Mahidol University, Nakhon Pathom 73170, Thailand; ^3^Department of Biochemistry, Faculty of Medicine, Chiang Mai University, Chiang Mai 50200, Thailand; ^4^Department of Medicine, Faculty of Medicine Siriraj Hospital, Mahidol University, Bangkok 10700, Thailand

## Abstract

Studies on the antioxidant treatment for thalassemia have reported variable outcomes. However, treatment of thalassemia with a combination of hydrophobic and hydrophilic antioxidants and an iron chelator has not been studied. This study investigated the effects of antioxidant cocktails for the treatment of *β*-thalassemia/hemoglobin E (HbE), which is the most common form of *β*-thalassemia in Southeast Asia. Sixty patients were divided into two groups receiving N-acetylcysteine, deferiprone, and either curcuminoids (CUR) or vitamin E (Vit-E), and their hematological parameters, iron load, oxidative stress, and blood coagulation potential were evaluated. Patients were classified as responders if they showed the improvements of the markers of iron load and oxidative stress, otherwise as nonresponders. During treatment, the responders in both groups had significantly decreased iron load, oxidative stress, and coagulation potential and significantly increased antioxidant capacity and hemoglobin concentration. The significantly maximum increase (*P* < 0.01) in hemoglobin concentration was 11% at month 4 in CUR group responders and 10% at month 10 in Vit-E group responders. In conclusion, the two antioxidant cocktails can improve anemia, iron overload, oxidative stress, and hypercoagulable state in *β*-thalassemia/HbE.

## 1. Introduction

*β*-thalassemia/hemoglobin E (HbE) (*β* ^0/+^/*β* ^E^) is the most common form of *β*-thalassemia in Southeast Asia [1]. In patients with *β*-thalassemia/HbE, partially or completely impaired synthesis of *β*-globins results in excess numbers of *α*-globins in mature and immature red blood cells (RBC). This leads to massive reactive oxygen species (ROS) formation via the Fenton reaction followed by oxidative damage mainly to the cell membrane [2]. Lipid peroxidation and phosphatidylserine (PS) externalization are involved in initiating and triggering of apoptosis resulting in ineffective erythropoiesis, premature hemolysis, and subsequent anemia [2]. PS-exposed RBCs and platelets also have platelet factor 3- (PF3-) like activity, resulting in activation of platelets and the coagulation mechanism [3, 4].

Oxidative stress is continuously exacerbated by secondary iron overload resulting from regular blood transfusions in transfusion-dependent patients and increased dietary iron absorption in transfusion-independent patients [5]. After transferrin is fully saturated, the excess iron binds to low-molecular-weight ligands forming non-transferrin-bound iron (NTBI) [6]. NTBI is potentially toxic because of its uncontrolled translocation across cell membranes and induction of ROS production [7]. Increased levels of ROS result in oxidative modification of biomolecules, leading to cellular toxicity and functional impairment of organs with NTBI deposition [8]. Oxidative stress and iron overload therefore play key roles in the pathophysiology of thalassemia and its complications [2].

Previous studies of antioxidant and chelation therapy for thalassemia reported variable efficacy in terms of the decrease in oxidative stress, improvement of antioxidant defense systems, and decrease in iron load [9–14]. Improvement of anemia after these therapies has not been reported. Since treatment of thalassemia with a combination of hydrophobic antioxidant, hydrophilic antioxidant, and iron chelator has not been studied, this clinical trial aimed at the evaluation of the effects of two antioxidant cocktails (each comprising a hydrophobic antioxidant, a hydrophilic antioxidant, and an iron chelator) on the iron load, oxidative stress, antioxidant status, blood coagulation potential, and anemia (especially the capability of raising hemoglobin (Hb) levels) in patients with *β*-thalassemia/HbE. Curcuminoids (CUR)/vitamin E (Vit-E), N-acetylcysteine (NAC), and deferiprone (DFP) were chosen to study because of the extensive studies on their efficiencies for the treatment of thalassemia [9, 13–18].

## 2. Methods

### 2.1. Patients, Interventions, and Blood Collection

The study was approved by the Siriraj Institutional Review Board, Mahidol University, and was registered with ClinicalTrials.gov (NCT01597765). *β*-thalassemia/HbE patients attended consultations at the Division of Hematology, Faculty of Medicine Siriraj Hospital, Mahidol University. Patients were screened, and 60 patients who met the inclusion criteria were enrolled. The inclusion criteria were genotype *β* ^0^/*β* ^E^, age between 18 and 50 years, Hb concentration 50–90 g/L, serum aspartate aminotransferase (AST) and alanine aminotransferase levels less than 3 times the upper limit of normal range, no medications other than folic acid during the preceding 3 months, and signed informed consent. To eliminate the impact of donor blood on the parameters evaluated, only non-transfusion-dependent patients were included. Pregnant or breastfeeding patients were excluded.

The subjects were divided into 2 groups (*n* = 30 each) using allocation concealment to prevent selection bias. Each group was treated with a different antioxidant cocktail for 12 months. The CUR group received 500 mg/day CUR, 200 mg/day NAC, and 50 mg/kg/day DFP, and the Vit-E group received 400 IU/day Vit-E, 200 mg/day NAC, and 50 mg/kg/day DFP. Two peripheral blood samples were collected 2 weeks apart from the measurement of baseline values, and additional samples were collected every 2 months during the treatment and at 3 months after the end of the treatment. Physical examinations were performed at the time of blood collection to record any adverse effects of the antioxidant cocktails and any medical issues related to the thalassemia.

The blood was stored in plain tubes and in tubes containing 3.2% trisodium citrate (for analysis of coagulation parameters), sodium heparin (for analysis of PS exposure), and K_3_EDTA. Serum from clotted blood was used for measurement of iron parameters and clinical chemistry tests. The complete blood count and ROS level were measured in EDTA blood. The remaining EDTA blood was centrifuged, and the packed red cells were washed, diluted to 50% hematocrit with cold phosphate buffered saline (pH 7.4), and kept at −80°C for the measurements of RBC MDA and antioxidant parameters.

Subjects were classified as responders or nonresponders based on the percentage changes in serum ferritin level (an indirect estimate of body iron burden) and RBC MDA concentration (a marker of oxidative stress) after 4 months of the treatment:

(1) Change (%)=month 4 value−baseline valuebaseline value×100.

Subjects with a >20% decrease in both serum ferritin and RBC MDA levels were classified as responders; otherwise they were classified as nonresponders.

### 2.2. Hematological Parameters and Clinical Chemistry Tests

The hematological parameters were analyzed using an automated hematology analyzer (Sysmex NE-1500; Sysmex, Tokyo, Japan). Liver function, renal function, and markers of hemolysis including serum AST, total bilirubin (TB), and indirect bilirubin (IDB) levels were analyzed using an automated Integra 700 analyzer (Roche Diagnostics, Basel, Switzerland).

### 2.3. Iron Parameters

The serum NTBI level was determined using the method of Singh et al. [19]. Nitrilotriacetic acid (NTA) was added to the serum to chelate ferric iron and formed Fe(III)-[NTA]_2_ complexes. The Fe(III)-[NTA]_2_ complex concentration was measured by reverse-phase high-performance liquid chromatography with 3-hydroxy-1-propyl-2-methylpyridine-4-one (CP22) on-column derivatization.

The serum ferritin level was determined using an automated chemical analyzer (Integra 700; Roche, Rotkreuz, Switzerland).

### 2.4. Oxidative Stress Parameters

The blood ROS levels were measured by staining fresh EDTA blood with 2′,7′-dichlorofluorescein diacetate (Sigma-Aldrich, St Louis, MO, USA). The cell-permeant dye reacts with intracellular ROS to form 2′,7′-dichlorofluorescein, which was detected using a FACSCalibur flow cytometer (Becton Dickinson Biosciences, Mountain View, CA, USA) [20].

The RBC MDA concentration was determined by the method of Stocks and Dormandy [21]. MDA formation was induced in RBC by adding hydrogen peroxide to 50% RBC suspension, followed by reaction with thiobarbituric acid in boiling conditions to form MDA-TBAR, which was measured by spectrophotometry at 532 and 600 nm.

### 2.5. Antioxidant Parameters

The measurement of superoxide dismutase (SOD) activity in 50% RBC suspension was based on the ability of SOD to inhibit the reduction of nitroblue tetrazolium by superoxide anions. The inhibitory activity of SOD was measured by a colorimetric method using a spectrophotometer at 560 nm [22].

Glutathione peroxidase (GPx) activity in 50% RBC suspension was indirectly determined by measuring the rate of NADPH oxidation in a coupled system containing* t*-butyl hydroperoxide, glutathione reductase, and glutathione (GSH) [23]. The rate of NADPH oxidation was measured by the decrease in absorbance at 340 nm using a spectrophotometer in kinetic mode.

The measurement of GSH levels in 50% RBC suspension was based on the reaction between the sulfhydryl group of GSH and 5,5′-dithiobis(2-nitrobenzoic acid) (DTNB) to form the stable yellow complex [24]. Briefly, the RBC suspension was first deproteinized and centrifuged to remove the precipitated proteins. Then, 250 *μ*L of filtrate was mixed and incubated for 5 minutes with 1 mL of 0.3 M phosphate solution and 125 *μ*L of DTNB reagent (deionized water used instead of DTNB as a blank) before measuring the absorbance at 412 nm. The difference of the absorbance between the sample with and without DTNB addition was used to calculate the GSH level.

### 2.6. Coagulation Parameters

PF3-like activity was determined using the method of Opartkiattikul et al. [3]. The intrinsic coagulation system was activated by addition of ellagic acid and calcium chloride to generate thrombin. The reaction between thrombin and its synthetic substrate S-2238, which forms a yellow compound that absorbs light at 405 nm, was measured.

The proportions of PS-exposed RBC (PS^+^RBC) and platelets (PS^+^Plt) were analyzed by flow cytometry [25]. Heparinized blood was stained using anti-human CD41a-FITC (Becton Dickinson Biosciences, San Jose, CA, USA), anti-human glycophorin A-PE (Dako, Glostrup, Denmark), and Annexin V-FITC (BD Pharmingen, San Diego, CA, USA). The samples were then analyzed using a FACSCalibur flow cytometer (Becton Dickinson Biosciences, Mountain View, CA, USA) and CellQuest software.

To measure the markers of platelet activation, platelets in citrated blood were activated by adenosine diphosphate and then labeled with anti-human CD41a-FITC, anti-CD62-FITC (Cymbus Biotechnology, Chandlers Ford, UK), and anti-PAC1-FITC (Becton, Dickinson and Co., Heidelberg, Germany). The CD62 and PAC1 concentrations were measured by flow cytometry [26].

Prothrombin time (PT) and activated partial thromboplastin time (aPTT) were measured in citrated blood using an automated Sysmex CA-1500 analyzer (Siemens, New York, NY, USA).

### 2.7. Statistical Analysis

Patient characteristics were analyzed using Chi-square test. Data from different time points were compared within the same treatment group using one-way analysis of variance followed by a least significant difference test, with *P* < 0.05 considered statistically significant. All analyses were performed using SPSS software, version 15.0 (SPSS Inc., Chicago, IL, USA).

## 3. Results

Out of 60 patients initially enrolled in the study, 50 (83.3%) patients completed the full 12 months of antioxidant treatment (25 in the CUR group and 25 in the Vit-E group). Ten patients discontinued the treatment because of accidental death (*n* = 1), loss to follow-up (*n* = 5), and receiving other essential medications (*n* = 4). The age, sex, and proportion of patients undergone splenectomy were not significantly different between the CUR and Vit-E groups (Table 1). No severe adverse effects related to the antioxidant treatments were observed. Mild adverse effects were reported in 8 patients: nausea/vomiting in 6 patients (4 in the CUR group and 2 in the Vit-E group), arthralgia in 1 patient in the CUR group, and rash in 1 patient in the Vit-E group. Using the criteria based on the percentage changes in serum ferritin and RBC MDA levels, 16 out of the 25 patients in the CUR group and 19 of the 25 patients in the Vit-E group were classified as responders (Table 1).

**Figure 1:**
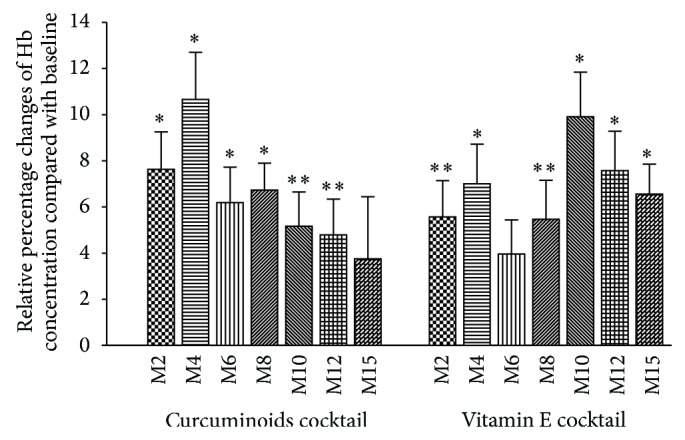
Percentage changes of hemoglobin concentration during and after the treatment period in patients with *β*-thalassemia/hemoglobin E who responded to treatment with antioxidant cocktails. The bars represent mean ± standard error of the mean. ^*∗*^ *P* < 0.01 compared with baseline, ^*∗∗*^ *P* < 0.05 compared with baseline.

In both responder groups, Hb concentration was significantly increased with nonsignificant trends towards an increase in RBC count and a decrease in reticulocyte count (Table 2). The maximum changes in the Hb concentration were approximately 11% at month 4 in the CUR responders and 10% at month 10 in the Vit-E responders (Figure 1). At month 15 (3 months after the end of the treatment), Hb concentrations in both groups were lower than the concentration at month 12 but still higher than at the baseline.

There were significant improvements of the markers of iron load (serum ferritin and NTBI levels) in the responders of both groups (Table 2). The ferritin levels decreased significantly to a minimum level at month 6 in the CUR group (*P* < 0.05) and at month 12 in the Vit-E group (*P* < 0.01), whereas the NTBI levels in the both groups decreased significantly in the first 6 months and remained unchanged afterwards.

Markers of oxidative stress (ROS levels and RBC MDA concentration) and antioxidant status (RBC SOD and GPx activities and GSH levels) were also improved (Table 2). In the CUR and Vit-E groups, responders had the lowest ROS level at month 6 and MDA at month 12. Increased levels of all markers of oxidative stress were observed after the end of the treatment.

In both groups, the responders had significantly decreased SOD and GPx activities (*P* < 0.01), significantly increased GSH level (*P* < 0.05 for the CUR group, *P* < 0.01 for the Vit-E group), and significantly decreased levels of markers of hemolysis including AST (*P* < 0.01 for the Vit-E group), TB (*P* < 0.05 for the CUR group), and IDB (*P* < 0.05 for the CUR group).

Responders to both antioxidant cocktails had progressively decreased proportions of PS^+^RBC and PS^+^Plt and decreased PF3-like activity during the treatment (procoagulation parameters), resulting in suppressed platelet activation shown by the significantly decreased CD62 and PAC1 expression (Table 3). The PT and aPTT in the CUR and Vit-E responder groups were close to the normal range (10.0–13.0 s for PT and 23.0–31.0 s for aPTT) indicating a decrease in coagulation potential. Most of the parameters analyzed returned to baseline values at month 15.

## 4. Discussion

One-year treatment of *β*-thalassemia/HbE with the CUR or Vit-E antioxidant cocktail was safe since no abnormalities in hematological parameters, liver function, or renal function were observed (data not shown). The mild adverse effects reported in this study are the most frequent side effects reported in patients taking DFP [27, 28]. None of the patients with the side effects discontinued the treatment. The severe side effects including granulocytosis or neutropenia were not observed in any of the patients. Compared to a previous study which treated thalassemic patients with DFP monotherapy at similar doses (25–50 mg/kg/day) [29], the patients in this study showed obviously lower incidences of side effects (gastrointestinal symptoms: 12% in antioxidant cocktails and 67% in DFP monotherapy; arthralgia: 2% in antioxidant cocktails and 12% in DFP monotherapy). These findings may suggest that the combination of an iron chelator with antioxidants may lessen its side effects more than taking only single DFP medication. However, long-term safety of the 2 antioxidant cocktails should be further investigated in a clinical trial with larger patient numbers and longer periods of treatment and follow-up.

Oxidative stress and iron overload are the main pathophysiological mechanisms in thalassemia. Previous studies reported higher levels of markers indicating iron overload and oxidative stress in thalassemic patients, compared with normal subjects [9, 30, 31]. During the treatment, the response to antioxidant cocktails varied considerably. For analysis, patients were classified as responders or nonresponders according to the improvements in iron load and oxidative stress during the first 4 months of treatment (Table 1). The molecular mechanisms contributing to response variation remain unknown. It is likely to involve genetic variations in antioxidant/iron chelator metabolism and lifestyle factors such as activities, pollution, and diet [32].

This study revealed decreases in the iron load, oxidative stress, antioxidant status, hypercoagulable state, and anemia in responders of both CUR and Vit-E groups. Therapeutic effects depend on the constituents of the cocktails. DFP is an iron chelator used orally in clinical practice that requires 3 molecules to fully bind with 1 iron atom (bidentate coordination) [33], while CUR chelate iron by direct binding to ferric ions via the *β*-diketone group and modulating the expression of proteins that induce iron depletion such as hepcidin, ferritin, transferrin receptor, and iron regulatory protein [34]. Responders to both cocktails reduced the iron load after 12 months of treatment, as shown by the significant reductions in serum ferritin (45% in CUR and 57% in Vit-E) and NTBI levels (62% in CUR and 63% in Vit-E) (Table 2).

Comparing our result with previous clinical trials is rather difficult because of the differences in criteria for subject recruitment, formulation, dose, and duration of administration. The previous study that used the same dose and treatment period of CUR monotherapy was compared [9], the ferritin levels were decreased by 45% with the CUR cocktail and 12% with the CUR monotherapy, and the NTBI levels decreased by 62% with the CUR cocktail and 15% with the CUR monotherapy at the end of treatment.

Comparing our results with a clinical trial that used the same dose of DFP monotherapy but different treatment period (7–17 months) [35], DFP monotherapy decreased the ferritin levels more than antioxidant cocktails (45% in CUR, 57% in Vit-E, and 73% in DFP monotherapy), but antioxidant cocktails decreased the NTBI levels more than DFP monotherapy (62% in CUR, 63% in Vit-E, and 49% in DFP monotherapy).

ROS in thalassemia are mainly generated by the iron-catalyzed Fenton reaction. Hence, the decrease in iron bioavailability reduced the formation of ROS. Besides preventing the formation, the generated ROS were eliminated by radical-scavenging activities of CUR (via *β*-diketone or methoxyl/hydroxyl groups on two phenolic sites [36]), Vit-E (hydroxyl group and electrons of the chromanol ring [37]), and NAC (sulfhydryl group [38]) in the cocktails. The chain-initiating radicals (such as alkyl and peroxyl radicals) were also scavenged by CUR and Vit-E to suppress lipid peroxidation [36, 37]. On the other hand, RBC antioxidant capacity was raised from the actions of NAC and CUR by increasing GSH levels. NAC provides cysteine and CUR increase the expression of *γ*-glutamylcysteine synthetase, which are the rate-limiting substrate and the rate-limiting enzyme of GSH synthesis, respectively [39, 40].

One of the major features of thalassemic RBC, PS externalization, is caused by oxidative membrane damage that leads to increased calcium influx, ATP depletion, and inactivation of ATP-dependent aminophospholipid translocase (APLT, the enzyme facilitating the PS translocation from the outer leaflet to the inner leaflet of the membrane) [41]. PS exposure promotes phagocytosis of erythroblasts and circulating RBC by reticuloendothelial cells [2]. Lower levels of PS^+^RBC, PS^+^Plt, and hemolysis markers in the responders may be the results of decreased oxidative stress as well as increased APLT activity by Vit-E [42].

As previously mentioned, RBC environment shifted towards a more reduced state in the responders with significantly decreased levels of ROS, oxidative product (MDA), antioxidant enzyme activities (SOD and GPx), and PS-exposed cells, accompanied by increased intracellular GSH levels. Compared with CUR monotherapy [9], administration of CUR cocktail resulted in greater reductions in RBC MDA (39% in CUR and 29% in CUR monotherapy) and GPx activity (43% in CUR and 22% in CUR monotherapy) after 12 months of treatment. Reduced SOD activity was comparable in patients treated with the CUR cocktail (12%) and with CUR monotherapy (14%).

This is the first report on an increase in Hb concentration after treatment with antioxidant cocktails in patients with non-transfusion-dependent *β*-thalassemia/HbE. The Hb concentration increased significantly in responders of both CUR and Vit-E groups. Responders also had an insignificant increase in RBC count and an insignificant decrease in reticulocyte count. These results may reflect improvement of RBC quality rather than stimulation of erythropoiesis. Decreased iron load and decreased oxidative stress ameliorate the harmful ROS-induced RBC membrane damage, resulting in less deformability, increased membrane stability, and inhibited PS externalization, thereby decreasing RBC destruction when passing through narrow capillaries and decreasing phagocytosis.

The maximum increase in Hb concentration occurred at different time points in the responders of both groups. The maximum increase in Hb concentration was approximately 11% at month 4 in the CUR responders and 10% at month 10 in the Vit-E responders. The late response in the Vit-E group may result from Vit-E deficiency in patients with thalassemia (the baseline serum Vit-E level ranged from 2 mg/L to 4 mg/L, data not shown). A previous study found that subjects with a serum Vit-E level below 5 mg/L had greater peroxide-dependent hemolysis than those with a serum Vit-E level above 5 mg/L [43]. Administration of Vit-E may replenish the body's reserves before the full antioxidant activity was available.

The hypercoagulable state improved in the responders of both groups. PS-exposed RBC and platelet membranes have PF3-like activity, inducing activation of platelets and the coagulation mechanism [44, 45]. Reduced PS exposure resulting from the treatment suppressed platelet activation as shown by decreased PF3-like activity and decreased CD62 and PAC1 expression. The antioxidants used in this study also have anticoagulant properties through other mechanisms. CUR decreases platelet adhesion, release reaction, and aggregation by inhibition of cyclooxygenase- and calcium-activated signaling cascades [46]. Vit-E decreases platelet adhesion/aggregation and platelet pseudopodia formation through the inhibition of protein kinase C [47]. NAC interrupts the formation of disulfide bonds in von Willebrand factor multimerization, a key event of the initiation of platelet aggregation [48]. The hypercoagulable state improved in the responders of both groups, as shown by the decreased PT and PT/aPTT ratio.

In summary, 12 months of treatment with both of the antioxidant cocktails including hydrophobic and hydrophilic antioxidants and an iron chelator significantly decreased iron loading and oxidative stress and improved the hypercoagulable state in patients with non-transfusion-dependent *β*-thalassemia/HbE. This is the first study to report an increase in Hb concentration after the treatment with antioxidant cocktails in patients with non-transfusion-dependent *β*-thalassemia/HbE. Treatment of patients with thalassemia and iron overload with the CUR cocktail is encouraged, because it may chelate iron and increase Hb concentration faster than with the Vit-E cocktail. However, this trial was limited by the small sample size from a single center. Our findings could be confirmed by the randomized controlled clinical trial with a larger sample size from multicenters. Moreover, future studies should identify prognostic predictors of treatment response to the antioxidant cocktails.

## Figures and Tables

**Table 1:** Patient characteristics.

	Curcuminoids cocktail			Vitamin E cocktail		
	Total	Responders	Non-responders	Total	Responders	Non-responders
Number of patients	25	16	9	25	19	6
Age (years)	32.5 ± 1.7	33.9 ± 2.5	30.1 ± 1.7	33.6 ± 2.1	33.0 ± 2.0	31.6 ± 4.3
Female : male, *n*	14 : 11	7 : 9	7 : 2	19 : 6	13 : 5	6 : 1
Splenectomy, *n*	11	7	4	9	6	3
Mean % change of serum ferritin at month 4 from baseline	−28.9	−39.7	+4.1	−33.5	−42.5	+4.7
Mean % change of RBC MDA at month 4 from baseline	−15.8	−24.2	−4.0	−30.1	−37.0	−5.4

The values represent mean ± standard error of the mean.

MDA: malondialdehyde; RBC: red blood cells.

**Table 2:** Hematological, iron load, oxidative stress, and antioxidant parameters and markers of hemolysis, in responders.

Parameters	Curcuminoids cocktail (*n* = 16)				Vitamin E cocktail (*n* = 19)			
	Baseline	Month 6	Month 12	Month 15	Baseline	Month 6	Month 12	Month 15
Hematological parameters								
Hemoglobin (g/L)	61.9 ± 2.3	67.8 ± 2.8^a^	66.9 ± 2.5^a^	63.9 ± 3.8	65.9 ± 3.4	69.6 ± 3.2	71.7 ± 3.5^a^	68.1 ± 3.0
RBC count (×10^12^ cells/L)	3.40 ± 0.16	3.51 ± 0.15	3.52 ± 0.18	3.26 ± 0.18	3.63 ± 0.22	3.66 ± 0.19	3.75 ± 0.1	3.71 ± 0.22
Reticulocyte count (proportion of 1)	0.06 ± 0.01	0.03 ± 0.01^a^	0.04 ± 0.01	0.06 ± 0.01	0.05 ± 0.01	0.04 ± 0.01	0.04 ± 0.01	0.07 ± 0.02
Iron load parameters								
Serum ferritin (pmol/L)	3651 ± 855	1921 ± 426^a^	2018 ± 434^a^	2415 ± 598	4767 ± 773	2339 ± 532^b^	2065 ± 655^b^	2765 ± 622^a^
Serum NTBI (*μ*mol/L)	5.3 ± 0.6	2.1 ± 0.2^b^	2.0 ± 0.5^b^	4.8 ± 1.0	4.9 ± 0.6	1.8 ± 0.3^b^	1.8 ± 0.3^b^	5.2 ± 0.7
Oxidative stress parameters								
ROS (%MCF)	51.1 ± 8.8	29.7 ± 3.3^a^	31.6 ± 6.8^a^	33.4 ± 4.8^a^	53.0 ± 7.0	28.6 ± 3.6^a^	33.5 ± 7.4	48.8 ± 15.1
RBC MDA (nmol/g Hb)	1542 ± 165	1150 ± 107^a^	934 ± 81^b^	1469 ± 151	1487 ± 138	815 ± 33^b^	698 ± 24^b^	1175 ± 79
Antioxidant parameters								
RBC SOD (U/g Hb)	5395 ± 278	4318 ± 179^b^	4727 ± 259	5094 ± 334	5051 ± 188	4245 ± 196^b^	4075 ± 219^b^	5097 ± 293
RBC GPx (U/g Hb)	63.7 ± 3.2	48.9 ± 1.9^b^	36.6 ± 1.4^b^	51.6 ± 2.8^b^	62.6 ± 2.6	48.9 ± 2.5^b^	36.6 ± 1.7^b^	47.3 ± 2.1^b^
RBC GSH (mmol/L)	1.74 ± 0.05	2.12 ± 0.06^a^	1.79 ± 0.14	1.76 ± 0.06	1.81 ± 0.04	2.10 ± 0.05^b^	2.04 ± 0.05^a^	1.82 ± 0.05
Markers of hemolysis								
AST (U/L)	43.3 ± 5.3	34.7 ± 3.8	35.5 ± 4.6	36.7 ± 4.0	46.7 ± 5.6	29.3 ± 1.9^b^	26.9 ± 2.6^b^	30.1 ± 3.0^a^
Total bilirubin (*μ*mol/L)	79.4 ± 10.2	58.0 ± 7.7^a^	62.2 ± 10.2	65.7 ± 6.8	71.6 ± 8.7	57.7 ± 6.3	60.0 ± 7.6	66.2 ± 8.2
Indirect bilirubin (*μ*mol/L)	69.3 ± 9.2	45.9 ± 7.2^a^	29.4 ± 0.6	50.3 ± 9.6	60.8 ± 8.4	45.4 ± 6.0	49.1 ± 7.4	54.9 ± 8.0

The values represent mean ± standard error of the mean.

AST: aspartate transaminase; GPx: glutathione peroxidase; GSH: reduced glutathione; MDA: malondialdehyde; NTBI: non-transferrin-bound iron; RBC: red blood cells; ROS: reactive oxygen species; SOD: superoxide dismutase.

^a^ *P* < 0.05 compared with baseline, ^b^ *P* < 0.01 compared with baseline.

**Table 3:** Procoagulation parameters, markers of platelet activation, and blood coagulation tests in responders.

Parameters	Curcuminoids cocktail (*n* = 16)				Vitamin E cocktail (*n* = 19)			
	Baseline	Month 6	Month 12	Month 15	Baseline	Month 6	Month 12	Month 15
Procoagulation parameters								
PF3-like activity (A_405_)	1.15 ± 0.12	0.86 ± 0.09	0.78 ± 0.10^a^	1.16 ± 0.16	1.24 ± 0.10	0.84 ± 0.08^b^	0.67 ± 0.06^b^	1.17 ± 0.12
PS^+^RBC (%)	4.49 ± 0.71	1.90 ± 0.36^b^	1.64 ± 0.44^b^	4.18 ± 0.79	5.41 ± 1.03	2.10 ± 0.65^a^	1.73 ± 0.71^b^	3.55 ± 1.28
PS^+^Plt (%)	1.02 ± 0.32	0.56 ± 0.16	0.40 ± 0.14	1.16 ± 0.32	0.61 ± 0.15	0.31 ± 0.06	0.24 ± 0.04^a^	0.61 ± 0.16
Platelet activation								
CD62 expression (%)	21.9 ± 4.0	9.0 ± 1.9^b^	13.8 ± 3.2	18.1 ± 3.9	16.9 ± 3.1	7.8 ± 2.1	12.3 ± 3.3	20.0 ± 4.6
PAC1 expression (%)	3.8 ± 1.0	0.9 ± 0.3^b^	2.0 ± 0.6^a^	2.4 ± 0.7	4.6 ± 1.1	1.1 ± 0.5^a^	2.7 ± 1.0	4.1 ± 1.5
Blood coagulation								
PT (s)	15.1 ± 0.2	14.0 ± 0.2^b^	14.4 ± 0.2	14.5 ± 0.4	14.9 ± 0.2	13.9 ± 0.2^b^	14.2 ± 0.2^b^	14.6 ± 0.2
aPTT (s)	31.3 ± 0.6	28.9 ± 0.6^a^	29.3 ± 0.8^a^	29.6 ± 0.8	30.8 ± 0.3	29.1 ± 0.4^b^	29.0 ± 0.5^b^	29.4 ± 0.5^a^
PT/aPTT	0.50 ± 0.01	0.47 ± 0.01^a^	0.49 ± 0.01	0.50 ± 0.01	0.50 ± 0.01	0.47 ± 0.01^a^	0.49 ± 0.01	0.49 ± 0.01

The values represent mean ± standard error of the mean.

aPTT: activated partial thromboplastin time; CD62: platelet surface P-selectin; PAC1: activated glycoprotein IIb/IIIa; PF3: platelet factor 3; Plt: platelet; PS^+^: phosphatidylserine-positive; PT: prothrombin time; RBC: red blood cells.

^a^ *P* < 0.05 compared with baseline, ^b^ *P* < 0.01 compared with baseline.
